# Nonlinear association between triglyceride-glucose index and risk of hyperuricemia in early-stage cardiovascular-kidney-metabolic syndrome: a cross-sectional study of United States population

**DOI:** 10.3389/fcvm.2025.1553957

**Published:** 2025-10-17

**Authors:** Xinyang Chen, Yan Liang

**Affiliations:** ^1^ Department of Critical Care Medicine, West China Hospital, Sichuan University, Chengdu, China; ^2^ West China School of Nursing, Sichuan University, Chengdu, China; ^3^ Department of Neurology, West China Hospital, Sichuan University, Chengdu, China

**Keywords:** triglyceride-glucose index (TyG), hyperuricemia, cardiovascular-kidney-metabolic syndrome (CKM), threshold effect, United States

## Abstract

**Objective:**

Cardiovascular-kidney-metabolic (CKM) syndrome represents a critical intersection of cardiovascular, renal, and metabolic disorders, emphasizing the importance of early risk stratification and intervention. The triglyceride-glucose (TyG) index, a surrogate marker of insulin resistance, has shown promise in predicting cardiometabolic risk. However, its association with hyperuricemia in early-stage CKM syndrome remains uncertain.

**Methods:**

This study analyzed data from 14,716 adult participants in the NHANES 2005–2018 dataset. A complex survey weight design and multiple imputation techniques were utilized to address missing data. The relationship between the TyG index and hyperuricemia was examined using generalized additive models and piecewise regression, with multivariable logistic regression adjusting for 14 potential confounders.

**Results:**

The TyG index demonstrated a significant positive association with hyperuricemia. Each unit increase in the TyG index was associated with a 62% higher risk of hyperuricemia (OR = 1.62, 95% CI: 1.45–1.81). A non-linear relationship was identified, with an inflection point at a TyG index of 9.50. Below this threshold, higher TyG index values were significantly associated with increased odds of hyperuricemia (OR = 2.18, 95% CI: 1.82–2.61), while above the threshold, the association became non-significant (OR = 0.79, 95% CI: 0.57–1.10). Subgroup analyses confirmed consistent associations across various demographic and clinical characteristics.

**Conclusions:**

The TyG index may serve as a valuable biomarker for identifying hyperuricemia risk in individuals with early-stage CKM syndrome, offering potential utility in clinical and public health settings. Further longitudinal studies are warranted to confirm these findings and assess the impact of TyG index-guided interventions on CKM syndrome progression.

## 1 Introduction

Cardiovascular-kidney-metabolic (CKM) syndrome reflects the complex interplay of cardiovascular, renal, and metabolic disorders, which often coexist and amplify each other's clinical burden. The increasing prevalence of CKM syndrome worldwide has underscored the need for early identification of risk factors and targeted interventions to curb its progression (1). Among the various metabolic abnormalities associated with CKM syndrome, hyperuricemia—a condition characterized by elevated serum uric acid levels—has garnered attention for its role in exacerbating cardiovascular and renal dysfunction (2–4). The relationship between hyperuricemia and insulin resistance (IR) is well-documented, with emerging evidence suggesting that hyperuricemia may both result from and contribute to metabolic derangements (5, 6). However, the mechanisms linking these conditions remain incompletely understood, particularly in the context of early-stage CKM syndrome.

The triglyceride-glucose (TyG) index, a reliable surrogate marker of IR (7), has gained traction in recent years for its predictive value in cardiometabolic disorders (8, 9). Studies have shown that higher TyG index values are associated with an increased risk of type 2 diabetes, hypertension, and cardiovascular diseases (10–12). However, while its utility in assessing cardiometabolic risk has been established, limited research has explored the relationship between the TyG index and hyperuricemia, particularly in populations with CKM syndrome. Investigating this relationship could offer valuable insights into the role of IR in CKM-related metabolic disturbances and inform early intervention strategies.

In this study, we focus on individuals in the early stages of CKM syndrome to evaluate the relationship between the TyG index and hyperuricemia. By utilizing data from the National Health and Nutrition Examination Survey (NHANES) 2005–2018 and employing advanced statistical techniques, we aim to identify non-linear associations and thresholds for hyperuricemia risk. This study is unique in targeting an underexplored population—those in the early stages of CKM syndrome—and provides evidence for the TyG index as a practical biomarker for early intervention. These findings contribute to the growing need for proactive approaches in CKM syndrome management, focusing on risk prediction and prevention during its earliest phases.

## 2 Methods

### 2.1 Study population

This cross-sectional study utilized data from the NHANES 2005–2018. NHANES employs a complex, multistage probability sampling design to collect nationally representative data through standardized questionnaires, physical examinations, and laboratory tests. From the NHANES 2005–2018 population, we excluded participants aged <20 years, pregnant women, individuals with undetermined CKM syndrome status due to incomplete data for key staging variables [missing measurements for body mass index (BMI), waist circumference, blood pressure, glucose parameters, lipid profiles, kidney function, or cardiovascular disease history], those with missing data for TyG index calculation (fasting triglycerides and glucose) or serum uric acid measurements, and participants with stage 4 CKM syndrome, as our study focused specifically on early-stage disease progression and intervention opportunities. The final analytic sample comprised adults with early-stage (0–3) CKM syndrome.

### 2.2 Definition of CKM syndrome early-stage

The staging of CKM Syndrome was comprehensively delineated according to the 2023 American Heart Association Presidential Advisory Statement (1, 13). Our study concentrated on early-stage CKM syndrome (stages 0–3): Stage 0 indicates the absence of CKM risk factors; Stage 1 is characterized by excess or dysfunctional adiposity; Stage 2 encompasses metabolic risk factors or chronic kidney disease (CKD); Stage 3 includes subclinical cardiovascular disease. Within this classification, very high-risk kidney disease (stages G4 or G5) and high cardiovascular disease risk predicted by the Framingham risk score were considered equivalent markers of subclinical cardiovascular pathology. Kidney function was estimated using the CKD-EPI equation to calculate glomerular filtration rate (eGFR) and staged according to Kidney Disease Improving Global Outcomes (KDIGO) guidelines. Given the research's focus on early disease progression, participants with established cardiovascular disease, end-stage kidney disease, or advanced metabolic complications (Stage 4) were excluded.

### 2.3 Exposure variable

The TyG index was calculated as ln[fasting triglycerides (mg/dl) × fasting glucose (mg/dl)/2] (14). Blood samples were collected after 8–12 h of fasting at NHANES mobile examination centers. Serum triglycerides were measured using enzymatic methods (GPO-PAP method, Roche Diagnostics), and glucose was measured using hexokinase method (Roche/Hitachi Cobas C311). The TyG index was analyzed both as a continuous variable and as a categorical variable divided into quartiles (Q1: 5.65–8.16, Q2: 8.16–8.58, Q3: 8.58–9.03, Q4: 9.03–12.84).

### 2.4 Outcome variable

Hyperuricemia was defined as serum uric acid ≥7.0 mg/dl in males or ≥6.0 mg/dl in females (15). Serum uric acid was measured using uricase-peroxidase method (Beckman Coulter UniCel DxC 800 Synchron) under standardized conditions. All samples were processed within 24 h of collection. Laboratory personnel were blinded to participants' exposure status to minimize measurement bias.

### 2.5 Covariates

Covariates included age (continuous, in years), sex (male or female), race/ethnicity (Non-Hispanic White, Non-Hispanic Black, Mexican American, and Other races), poverty-income ratio (PIR) (categorized as low [<1.3], medium [1.3–3.5], and high [>3.5]), education level (less than high school, high school graduate, and more than high school), physical activity (METs/week, categorized as low [<600], moderate [600–1199], and vigorous [≥1200]), smoking status (never [<100 cigarettes lifetime], former [>100 cigarettes but stopped], current [>100 cigarettes and still smoking]), drinking status (heavy [≥3 drinks/day for women, ≥4 for men, or ≥5 binge drinking days/month], moderate [≥2 drinks/day for women, ≥3 for men, or twice monthly binge drinking], mild [all other cases]), BMI (continuous, in kg/m^2^), eGFR (continuous, in ml/min/1.73 m^2^, calculated using CKD-EPI equation), glucose metabolism state (normoglycemia, prediabetes, diabetes), hypertension (yes/no), hyperlipidemia (yes/no), and healthy eating index (HEI)-2015 score, range 0–100) (16, 17).

### 2.6 Statistical analysis

Baseline characteristics of participants across quartiles of the TyG were summarized using descriptive statistical methods. Continuous variables were presented as survey-weighted mean values with 95% confidence intervals, and categorical variables were presented as survey-weighted percentage proportions with 95% confidence intervals. Between-group differences were assessed using survey-weighted linear regression analysis for continuous variables and survey-weighted chi-square statistical tests for categorical variables.

Missing data in covariates were handled using Multiple Imputation by Chained Equations (MICE) to reduce bias and enhance analytical robustness. The imputation model incorporated all relevant predictors and outcome variables to preserve relationships between variables. Predictive mean matching (PMM) was used for continuous variables, logistic regression for binary variables, and multinomial regression for categorical variables, with 5 iterations to ensure convergence.

The association between the TyG index and hyperuricemia was examined using a three-step analytical approach. First, survey-weighted logistic regression models were constructed to evaluate the relationship. Three models were developed: Model 1, unadjusted; Model 2, adjusted for age, sex, and race/ethnicity; and Model 3, adjusted for a comprehensive set of covariates including age, sex, race/ethnicity, PIR, educational level, physical activity (METs/week), smoking status, drinking status, BMI, eGFR, glucose metabolism state, hypertension, hyperlipidemia, and HEI-2015. Second, generalized additive models and smooth curve fitting were employed to explore potential non-linear relationships between the TyG index and hyperuricemia. When non-linearity was identified, a recursive algorithm was used to calculate the inflection point, followed by the construction of survey-weighted piecewise logistic regression models for the segments on either side of the inflection point. The log-likelihood ratio test was applied to compare the standard logistic regression model with the piecewise model to determine the better fit. Finally, subgroup analyses were performed using survey-weighted stratified logistic regression models, stratified by variables such as age, sex, race, smoking status, alcohol consumption and eGFR. Continuous stratification variables were categorized based on clinically relevant cut-points before conducting interaction tests. Effect modification was assessed using likelihood ratio tests to evaluate interactions between variables and the TyG index.

For sensitivity analysis, we converted the TyG index into a categorical variable and calculated P for trend to verify the continuous variable analysis results and examine potential non-linearity. All analyses incorporated sampling weights, stratification, and cluster variables following NHANES analytical guidelines to account for the complex survey design and ensure nationally representative estimates. Statistical analyses were performed using R (version 4.2.2, http://www.R-project.org) and EmpowerStats (version 4.2, https://www.empowerstats.com). A two-sided *P* value <0.05 was considered statistically significant.

## 3 Results

### 3.1 Study sample selection

**Figure 1:**
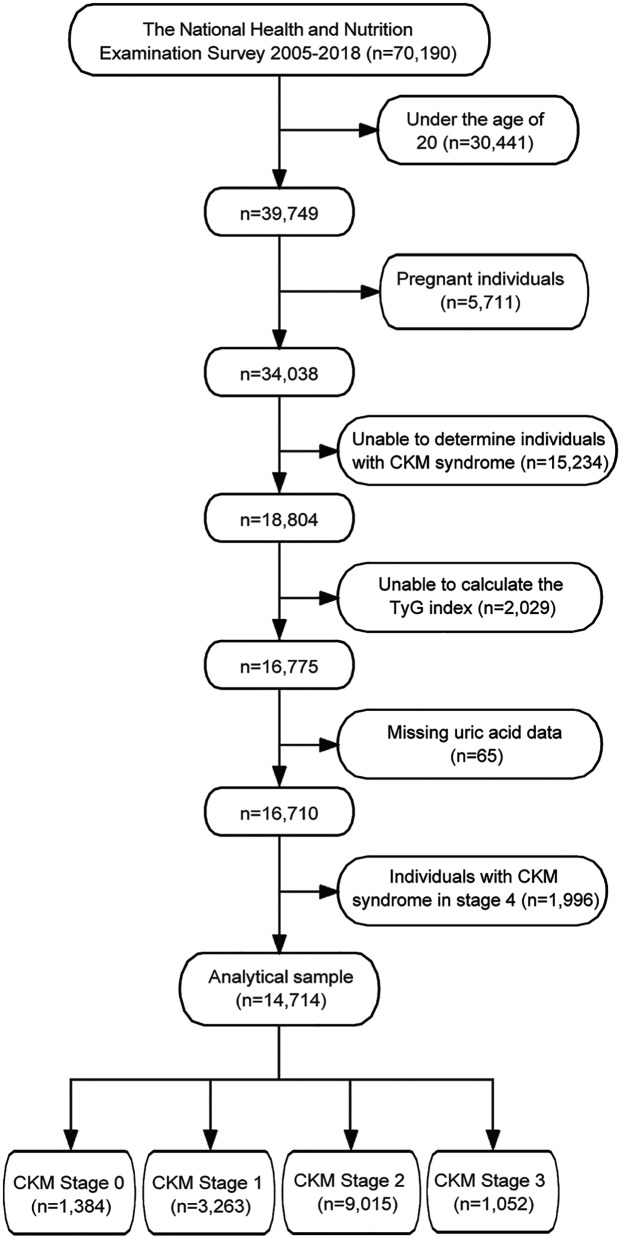
Flowchart of participant selection for the study. TyG, triglyceride-glucose; CKM, cardiovascular-kidney-metabolic.

Among the 70,190 participants from the NHANES (2005–2018), a total of 14,716 participants were included in the final analysis after applying the exclusion criteria (Figure 1). The exclusion process sequentially removed participants who were under 20 years old (*n* = 30,441), pregnant (*n* = 2,711), unable to determine CKM syndrome status (*n* = 8,234), missing TyG index data (*n* = 2,229), and missing uric acid measurements (*n* = 45). Additionally, 1,996 individuals with CKM syndrome in stage 4 were excluded from the analysis.

CKM syndrome stage distribution among the final study population (*n* = 14,714) was as follows: Stage 0 (*n* = 1,384, 9.4%), Stage 1 (*n* = 3,263, 22.2%), Stage 2 (*n* = 9,015, 61.3%), and Stage 3 (*n* = 1,052, 7.1%). Detailed CKM staging criteria are provided in Supplementary Table S1.

### 3.2 Baseline demographic characteristics

The baseline characteristics of 14,714 participants stratified by TyG index quartiles demonstrated significant differences across multiple domains (Table 1). In terms of demographic characteristics, mean age progressively increased from 41.29 years in Q1 to 50.10 years in Q4 (*P* < 0.001). Male proportion showed an ascending trend from 38.21% to 57.97% across quartiles (*P* < 0.001). Racial distribution varied significantly (*P* < 0.001), with Non-Hispanic Whites maintaining relatively stable proportions (63.43% to 68.51%), while Non-Hispanic Blacks decreased (16.54% to 5.64%) and Mexican Americans increased (6.51% to 11.42%). Higher education levels decreased from 68.57% to 54.81% (*P* < 0.001), while poverty income ratio showed no significant differences across quartiles (*P* = 0.083).

**Table 1:** Weighted baseline characteristics of study participants according to TyG index quartiles.

Variables	TyG index quartiles				*P*-value
	Q1 (5.65–8.16) *n* = 3,879	Q2 (8.16–8.58) *n* = 3,721	Q3 (8.58–9.03) *n* = 3,645	Q4 (9.03–12.84) *n* = 3,469	
Age (years)	41.29 (40.43, 42.15)	46.34 (45.59, 47.09)	48.34 (47.55, 49.13)	50.10 (49.44, 50.76)	<0.001
Sex (%)					<0.001
Male	38.21 (36.29, 40.17)	47.68 (45.80, 49.57)	52.26 (50.24, 54.28)	57.97 (55.75, 60.15)	
Female	61.79 (59.83, 63.71)	52.32 (50.43, 54.20)	47.74 (45.72, 49.76)	42.03 (39.85, 44.25)	
Race/ethnicity (%)					<0.001
Non-Hispanic White	63.43 (60.40, 66.35)	68.29 (65.36, 71.09)	67.77 (64.78, 70.63)	68.51 (65.29, 71.56)	
Non-Hispanic Black	16.54 (14.51, 18.79)	10.91 (9.58, 12.40)	7.64 (6.57, 8.87)	5.64 (4.79, 6.64)	
Mexican American	6.51 (5.32, 7.94)	8.26 (6.90, 9.85)	10.05 (8.54, 11.79)	11.42 (9.78, 13.30)	
Others	13.53 (11.75, 15.53)	12.54 (11.05, 14.20)	14.54 (13.02, 16.21)	14.43 (12.38, 16.75)	
PIR (%)					0.083
Low	20.49 (18.58, 22.55)	20.44 (18.73, 22.25)	21.40 (19.37, 23.58)	22.70 (20.81, 24.70)	
Medium	35.52 (33.36, 37.73)	35.06 (32.42, 37.78)	37.66 (35.10, 40.29)	37.08 (35.08, 39.11)	
High	43.99 (41.28, 46.73)	44.51 (41.10, 47.97)	40.94 (38.12, 43.83)	40.23 (37.73, 42.78)	
Education level (%)					<0.001
Less than high school	11.37 (10.06, 12.84)	14.70 (13.18, 16.36)	18.18 (16.37, 20.14)	19.88 (18.12, 21.77)	
High school	20.06 (18.23, 22.02)	23.27 (21.38, 25.28)	23.47 (21.44, 25.63)	25.31 (23.04, 27.72)	
More than high school	68.57 (65.84, 71.18)	62.03 (59.24, 64.73)	58.35 (55.58, 61.07)	54.81 (52.02, 57.58)	
METs/week (%)					<0.001
Low	19.54 (17.91, 21.27)	23.43 (21.81, 25.12)	25.32 (23.55, 27.17)	26.23 (24.50, 28.04)	
Moderate	2.34 (1.80, 3.05)	2.76 (2.09, 3.64)	2.48 (1.96, 3.14)	2.51 (1.89, 3.32)	
Vigorous	78.12 (76.36, 79.78)	73.81 (72.09, 75.46)	72.20 (70.22, 74.09)	71.26 (69.31, 73.13)	
Smoking (%)					<0.001
Never	63.58 (61.16, 65.93)	56.36 (53.75, 58.93)	54.42 (52.08, 56.74)	47.44 (45.13, 49.76)	
Former	20.05 (18.07, 22.19)	23.64 (21.39, 26.06)	24.88 (22.88, 26.99)	28.71 (26.51, 31.02)	
Now	16.37 (14.90, 17.96)	20.00 (17.92, 22.25)	20.70 (18.89, 22.64)	23.85 (21.95, 25.85)	
Drinking (%)					<0.001
Never	11.89 (10.60, 13.32)	10.72 (9.52, 12.05)	10.27 (9.09, 11.58)	11.59 (10.13, 13.24)	
Former	9.07 (8.02, 10.25)	11.23 (10.00, 12.59)	13.20 (11.89, 14.62)	15.69 (14.03, 17.50)	
Mild	36.96 (34.41, 39.58)	37.37 (35.05, 39.76)	39.13 (36.98, 41.33)	34.91 (32.42, 37.48)	
Moderate	21.90 (20.18, 23.72)	17.78 (16.06, 19.65)	15.59 (13.92, 17.42)	15.17 (13.50, 17.00)	
Heavy	20.18 (18.70, 21.74)	22.89 (21.09, 24.80)	21.81 (19.93, 23.82)	22.64 (20.65, 24.78)	
BMI (kg/m^2^)	26.17 (25.85, 26.50)	28.22 (27.91, 28.53)	29.97 (29.66, 30.28)	31.73 (31.37, 32.08)	<0.001
Height (cm)	168.18 (167.76, 168.60)	169.13 (168.75, 169.51)	168.84 (168.40, 169.29)	169.64 (169.16, 170.11)	<0.001
SBP (mmHg)	115.49 (114.82, 116.16)	119.81 (119.06, 120.56)	122.06 (121.34, 122.79)	126.28 (125.48, 127.08)	<0.001
DBP (mmHg)	67.73 (67.22, 68.25)	69.62 (69.07, 70.17)	71.04 (70.42, 71.67)	73.17 (72.65, 73.70)	<0.001
eGFR (ml/min/1.73 m^2^)	101.88 (100.87, 102.90)	96.06 (95.01, 97.11)	94.34 (93.35, 95.33)	93.21 (92.27, 94.16)	<0.001
Glucose metabolism state (%)					<0.001
Normoglycemia	88.08 (86.72, 89.33)	77.27 (75.56, 78.88)	63.50 (61.10, 65.84)	41.27 (38.83, 43.75)	
Prediabetes	8.52 (7.42, 9.77)	15.39 (13.90, 17.00)	22.30 (20.20, 24.56)	24.59 (22.94, 26.31)	
Diabetes	3.40 (2.77, 4.15)	7.35 (6.46, 8.35)	14.19 (12.80, 15.72)	34.15 (31.96, 36.40)	
Hypertension (%)					<0.001
No	79.63 (77.59, 81.53)	68.46 (66.14, 70.70)	60.31 (58.44, 62.16)	49.59 (47.15, 52.03)	
Yes	20.37 (18.47, 22.41)	31.54 (29.30, 33.86)	39.69 (37.84, 41.56)	50.41 (47.97, 52.85)	
Hyperlipidemia (%)					<0.001
No	61.37 (59.50, 63.21)	35.49 (33.40, 37.65)	18.07 (16.32, 19.96)	1.72 (1.22, 2.41)	
Yes	38.63 (36.79, 40.50)	64.51 (62.35, 66.60)	81.93 (80.04, 83.68)	98.28 (97.59, 98.78)	
HEI-2015	53.16 (52.57, 53.75)	51.96 (51.23, 52.70)	50.98 (50.36, 51.61)	50.83 (50.14, 51.51)	<0.001
TyG index	7.82 (7.81, 7.83)	8.38 (8.38, 8.39)	8.80 (8.79, 8.80)	9.49 (9.47, 9.51)	<0.001
Uric acid (mg/dl)	4.88 (4.82, 4.93)	5.33 (5.27, 5.39)	5.68 (5.62, 5.74)	6.00 (5.94, 6.06)	<0.001
Hyperuricemia (%)					<0.001
No	91.95 (90.55, 93.16)	86.20 (84.50, 87.73)	79.11 (77.28, 80.83)	69.88 (67.73, 71.94)	
Yes	8.05 (6.84, 9.45)	13.80 (12.27, 15.50)	20.89 (19.17, 22.72)	30.12 (28.06, 32.27)	
CKM syndrome stage (%)					<0.001
0	28.34 (26.46, 30.29)	12.26 (10.74, 13.95)	2.69 (2.13, 3.39)	0.00 (0.00, 0.00)	
1	36.82 (34.65, 39.04)	38.83 (36.50, 41.22)	16.68 (15.17, 18.30)	0.00 (0.00, 0.00)	
2	32.90 (30.75, 35.13)	45.22 (42.70, 47.76)	76.04 (74.26, 77.72)	92.69 (91.67, 93.60)	
3	1.94 (1.58, 2.36)	3.69 (3.13, 4.36)	4.60 (3.87, 5.46)	7.31 (6.40, 8.33)	

For continuous variables, data are presented as survey-weighted means (95% CI), and *P*-values were calculated using survey-weighted linear regression (svyglm). For categorical variables, data are presented as survey-weighted percentages (95% CI), and *P*-values were calculated using the survey-weighted Chi-square test (svytable).

TyG, triglyceride-glucose; PIR, poverty income ratio; MET, metabolic equivalent; BMI, body mass index; SBP, systolic blood pressure; DBP, diastolic blood pressure; eGFR, estimated glomerular filtration rate; HEI, healthy eating index; CKM, cardiovascular-kidney-metabolic syndrome.

Lifestyle characteristics demonstrated significant variations across TyG quartiles. Physical activity patterns showed decreased vigorous activity (78.12% to 71.26%) and increased low-intensity activity (19.54% to 26.23%) (*P* < 0.001). The proportion of never-smokers decreased from 63.58% to 47.44%, while current smokers increased from 16.37% to 23.85% (*P* < 0.001). Among drinking patterns, former drinkers increased from 9.07% to 15.69%, while moderate drinkers decreased from 21.90% to 15.17% (*P* < 0.001).

Clinical parameters showed consistent trends of deterioration with increasing TyG index. BMI increased from 26.17 to 31.73 kg/m^2^ (*P* < 0.001), accompanied by elevated blood pressure (SBP: 115.49 to 126.28 mmHg; DBP: 67.73 to 73.17 mmHg; both *P* < 0.001). Kidney function, assessed by eGFR, declined from 101.88 to 93.21 ml/min/1.73 m^2^ (*P* < 0.001), while HEI-2015 scores decreased from 53.16 to 50.83 (*P* < 0.001).

Metabolic parameters demonstrated substantial deterioration across quartiles. The prevalence of normoglycemia decreased markedly from 88.08% to 41.27%, while diabetes increased from 3.40% to 34.15% (*P* < 0.001). Hypertension prevalence rose from 20.37% to 50.41%, and hyperlipidemia showed a dramatic increase from 38.63% to 98.28% (both *P* < 0.001). Mean TyG index progressed from 7.82 to 9.49 (*P* < 0.001). Serum uric acid levels increased from 4.88 to 6.00 mg/dl, with hyperuricemia prevalence rising from 8.05% to 30.12% (*P* < 0.001).

The distribution of CKM syndrome stages showed significant shifts across TyG quartiles (*P* < 0.001). Stage 0 decreased from 28.34% to 0% in Q4, while stage 2 became predominantly prevalent, increasing from 32.90% to 92.69%. Stage 3 showed a gradual rise from 1.94% to 7.31%, indicating a progressive worsening of cardiometabolic health with increasing TyG index.

### 3.3 Association between TyG index and hyperuricemia in early-stage CKM syndrome

In the weighted analysis examining the association between TyG index and hyperuricemia among participants with early-stage CKM syndrome, both continuous and categorical analyses revealed significant associations (Table 2). When analyzed as a continuous variable, each unit increase in TyG index was associated with 2.13-fold (95% CI: 1.96–2.31) increased odds of hyperuricemia in the unadjusted model, which remained largely unchanged after adjusting for demographic factors (OR = 2.11, 95% CI: 1.94–2.30) and was attenuated but remained significant after full adjustment for potential confounders (OR = 1.62, 95% CI: 1.45–1.81). In the quartile analysis, compared with the lowest TyG index quartile (Q1: 5.65–8.16), the fully adjusted odds ratios for hyperuricemia were 1.41 (95% CI: 1.11–1.80) for Q2 (8.16–8.58), 2.00 (95% CI: 1.58–2.55) for Q3 (8.58–9.03), and 2.91 (95% CI: 2.25–3.76) for Q4 (9.03–12.84), with significant trends across quartiles (P for trend <0.001) in all models.

**Table 2:** Weighted analysis of the association between TyG index and hyperuricemia in a population with early-stage (stages 0–3) CKM syndrome.

TyG index	Model 1	Model 2	Model 3
Continuous	2.13 (1.96, 2.31)	2.11 (1.94, 2.30)	1.62 (1.45, 1.81)
Quartiles			
Q1 (5.65–8.16)	Reference	Reference	Reference
Q2 (8.16–8.58)	1.83 (1.46, 2.30)	1.79 (1.42, 2.26)	1.41 (1.11, 1.80)
Q3 (8.58–9.03)	3.02 (2.46, 3.71)	2.98 (2.41, 3.68)	2.00 (1.58, 2.55)
Q4 (9.03–12.84)	4.92 (4.06, 5.97)	4.85 (3.96, 5.94)	2.91 (2.25, 3.76)
P for trend	<0.001	<0.001	<0.001

**Model 1:** Non-adjusted.

**Model 2:** Adjusted for age, sex, and race/ethnicity.

**Model 3:** Adjusted for age, sex, race/ethnicity, PIR, educational level, METs/week, smoking, drinking, BMI, eGFR, glucose metabolism state, hypertension, hyperlipidemia, and HEI-2015.

TyG, triglyceride-glucose; CKM, cardiovascular-kidney-metabolic; PIR, poverty income ratio; MET, metabolic equivalent; BMI, body mass index; eGFR, estimated glomerular filtration rate; HEI, healthy eating index.

### 3.4 Threshold effect analysis of TyG index on hyperuricemia in early-stage CKM syndrome

**Figure 2:**
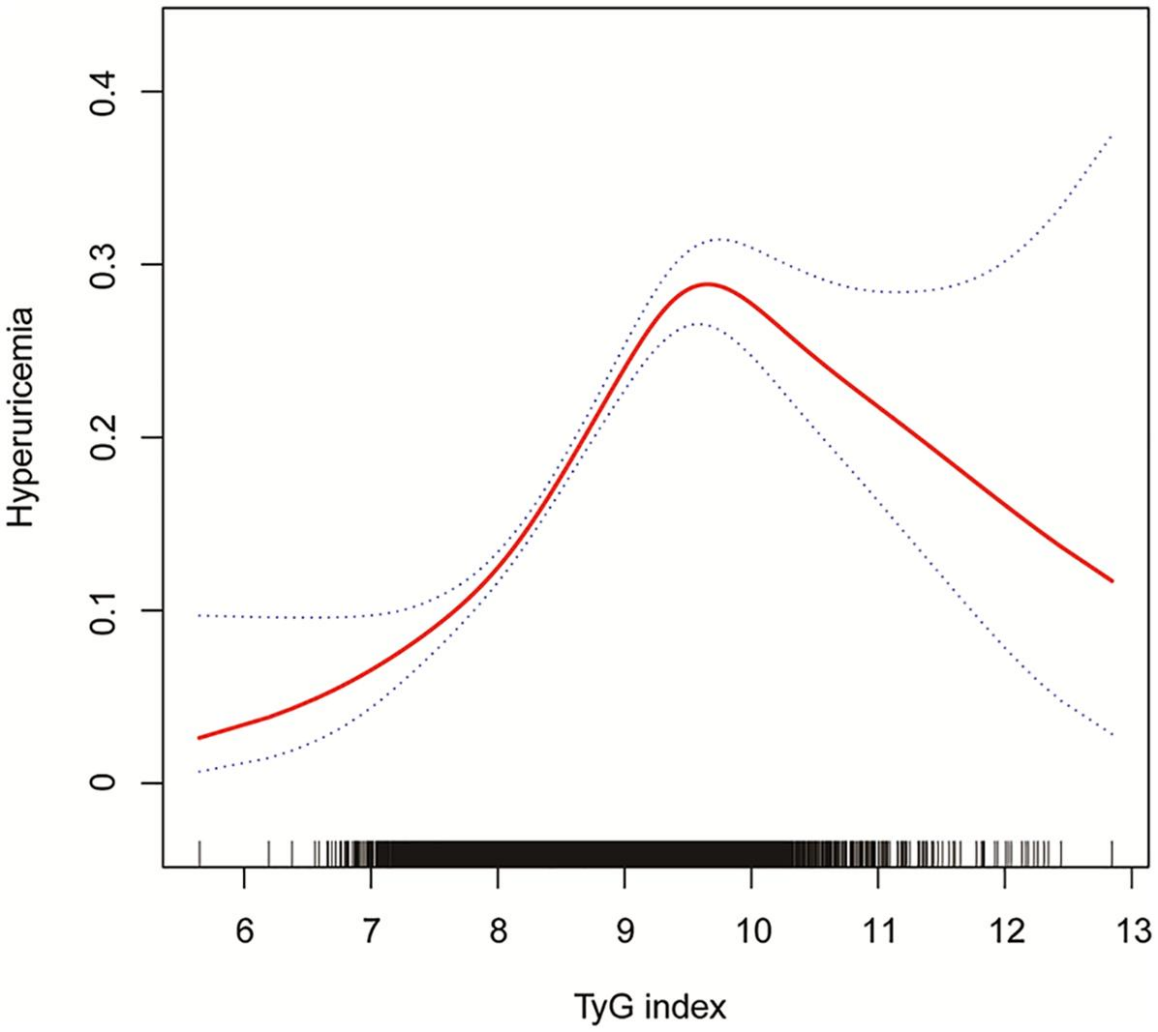
The association between TyG index and hyperuricemia in a population with early-stage (stages 0–3) CKM syndrome. Age, sex, race/ethnicity, PIR, educational level, METs/week, smoking, drinking, BMI, eGFR, glucose metabolism state, hypertension, hyperlipidemia, and HEI-2015 were adjusted. TyG, triglyceride-glucose; CKM, cardiovascular-kidney-metabolic; PIR, poverty income ratio; MET, metabolic equivalent; BMI, body mass index; eGFR, estimated glomerular filtration rate; HEI, healthy eating index.

Using GAM and smooth curve fitting, Figure 2 illustrated a nonlinear relationship between TyG index and hyperuricemia. The smooth curve demonstrated an initial positive association with increasing TyG index values, followed by a declining trend after reaching a peak at approximately TyG index of 9–10. This visual representation of the nonlinear pattern aligned with the threshold effect subsequently quantified by two-piecewise logistic regression analysis in Table 3.

**Table 3:** Weighted two-piecewise logistic regression analysis of the association between TyG index and hyperuricemia in a population with early-stage (stages 0–3) CKM syndrome.

TyG index	Adjusted OR^a^ (95% CI)	*P*-value
Model I		
Fitting by the standard linear model	1.62 (1.45, 1.81)	<0.001
Model II		
Inflection point	9.50	
<9.50	2.18 (1.82, 2.61)	<0.001
>9.50	0.79 (0.57, 1.10)	0.164
Log likelihood ratio	/	<0.001

TyG, triglyceride-glucose; CKM, Cardiovascular-Kidney-Metabolic; OR, odds ratio; CI, confidence interval; PIR, poverty income ratio; MET, metabolic equivalent; BMI, body mass index; eGFR, estimated glomerular filtration rate; HEI, healthy eating index.

^a^ Adjusted for age, sex, race/ethnicity, PIR, educational level, METs/week, smoking, drinking, BMI, eGFR, glucose metabolism state, hypertension, hyperlipidemia, and HEI-2015.

Two-piecewise logistic regression analysis revealed a nonlinear relationship between TyG index and hyperuricemia in early-stage CKM syndrome (Table 3). While the standard linear model showed that each unit increase in TyG index was associated with 1.62-fold increased odds of hyperuricemia (95% CI: 1.45–1.81, *P* < 0.001), further threshold effect analysis identified an inflection point at TyG index of 9.50. Below this threshold, each unit increase in TyG index was associated with significantly higher odds of hyperuricemia (OR = 2.18, 95% CI: 1.82–2.61, *P* < 0.001). However, beyond the threshold of 9.50, this association was no longer significant (OR = 0.79, 95% CI: 0.57–1.10, *P* = 0.164). The log likelihood ratio test (*P* < 0.001) supported the superior fit of the two-piecewise model over the linear model, suggesting a threshold effect in the association between TyG index and hyperuricemia.

### 3.5 Stratified analysis of the association between TyG index and hyperuricemia in early-stage CKM syndrome

**Figure 3:**
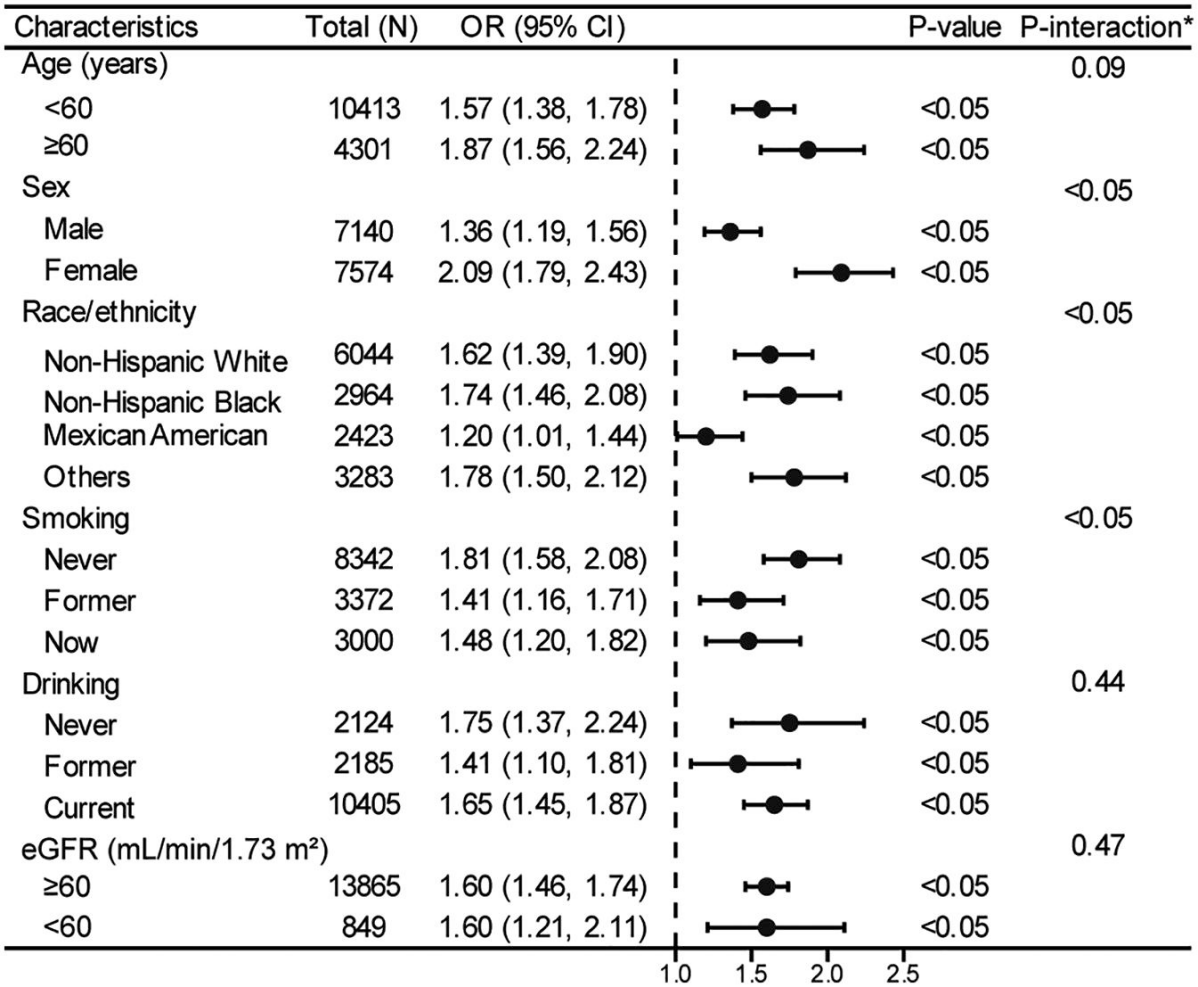
Stratified analyses between TyG index and hyperuricemia in a population with early-stage (stages 0–3) CKM syndrome. *Each stratification adjusted for all the factors (age, sex, race/ethnicity, PIR, educational level, METs/week, smoking, drinking, BMI, eGFR, glucose metabolism state, hypertension, hyperlipidemia, and HEI-2015) except the stratification factor itself. OR, odds ratio; CI, confidence interval; TyG, triglyceride-glucose; CKM, cardiovascular-kidney-metabolic; PIR, poverty income ratio; MET, metabolic equivalent; BMI, body mass index; eGFR, estimated glomerular filtration rate; HEI, healthy eating index.

In the stratified logistic regression analyses (Figure 3), significant interaction effects were observed for sex, race/ethnicity, and smoking status (all P-interaction < 0.05), while age groups, drinking status, and eGFR showed no significant interaction effects. Notably, despite these interaction differences, all subgroup analyses demonstrated statistically significant positive associations (all *P* < 0.05, OR > 1.0), further validating the robust relationship between TyG index and hyperuricemia established in our primary analysis (Table 2).

**Figure 4:**
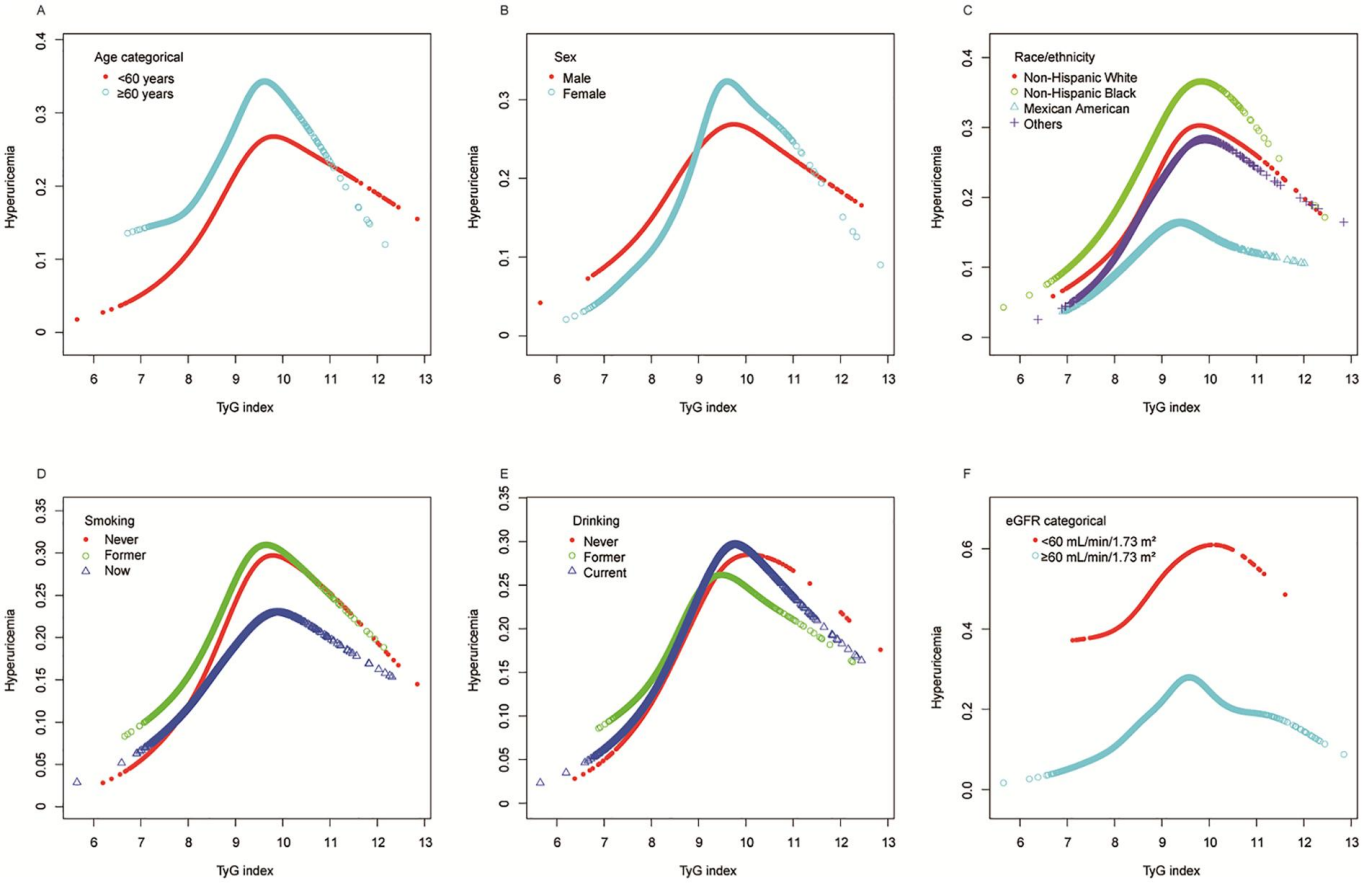
Stratified analyses (by **(A)** age; **(B)** sex; **(C)** race/ethnicity; **(D)** smoking; **(E)** drinking; **(F)** eGFR) between TyG index and hyperuricemia in a population with early-stage (stages 0–3) CKM syndrome using generalized additive model and smooth curve fittings. *Each generalized additive model and smooth curve fitting was adjusted for all factors, including age, sex, race/ethnicity, PIR, educational level, METs/week, smoking, drinking, BMI, eGFR, glucose metabolism state, hypertension, hyperlipidemia, and HEI-2015, except for the stratification factor itself. TyG, triglyceride-glucose; CKM, cardiovascular-kidney-metabolic; PIR, poverty income ratio; MET, metabolic equivalent; BMI, body mass index; eGFR, estimated glomerular filtration rate; HEI, healthy eating index.

Further stratified analyses using generalized additive models and smooth curve fitting (Figure 4) consistently revealed nonlinear relationships between TyG index and hyperuricemia across all subgroups. Although the specific patterns varied among different stratifications of age, sex, race/ethnicity, smoking status, drinking status and eGFR, the presence of nonlinearity remained evident throughout all analyses, providing additional support for the nonlinear association pattern identified in our main analysis (Figure 2).

## 4 Discussion

This study is the first to investigate the association between the TyG index and hyperuricemia in individuals with early-stage CKM syndrome. While previous studies have primarily focused on specific disease populations or high-risk groups, evidence from early-stage CKM syndrome population remains scarce. Using data from 14,716 participants with early-stage CKM syndrome in NHANES 2005–2018, with complex survey design methods, we ensured accurate and nationally representative estimates. After comprehensive adjustment for potential confounders, each unit increase in TyG index was associated with 62% higher odds of hyperuricemia (OR = 1.62, 95% CI: 1.45–1.81). Notably, generalized additive model analysis revealed a non-linear relationship with an inflection point at TyG index of 9.50: below this threshold, each unit increase in TyG index was associated with 118% higher odds of hyperuricemia (OR = 2.18, 95% CI: 1.82–2.61), while beyond this point, the association reversed to negative and became statistically non-significant (OR = 0.79, 95% CI: 0.57–1.10). These findings provide important implications for hyperuricemia risk assessment in early-stage CKM syndrome population.

The relationship between IR and hyperuricemia has garnered considerable attention in recent years. Studies have demonstrated that IR may influence uric acid metabolism and excretion through multiple mechanisms, thereby promoting the development of hyperuricemia. First, IR is closely associated with the renal handling of uric acid. Research indicates that IR leads to increased tubular reabsorption of uric acid, reducing its excretion and subsequently elevating serum uric acid levels (18). Furthermore, IR is also linked to reduced renal sodium excretion, which may further impact uric acid metabolism (19). Second, IR is intricately related to other components of metabolic syndrome, such as obesity and hypertension. Both obesity and hypertension are independent risk factors for hyperuricemia, and IR plays a pivotal role in the development and progression of these conditions (11, 20). Consequently, IR may indirectly contribute to hyperuricemia by influencing these metabolic disturbances. Although the hyperinsulinemic-euglycemic clamp (HEC) remains the gold standard for assessing IR, its technical complexity and high cost render it impractical for routine clinical use (21). The TyG index has emerged as a reliable surrogate marker for IR due to its simplicity, ease of calculation, and potential for widespread clinical application.

Consistent with the findings of this study, multiple research studies provide consistent evidence of a positive correlation between the TyG index and the risk of hyperuricemia. In a comprehensive analysis involving 30,453 individuals aged 50 and older, it was found that for each unit increase in the TyG index, the risk of hyperuricemia increased by 1.44 times in men and by 1.69 times in women, even after adjusting for confounding factors (22). Another study involving 14,286 American adults and 4,620 Chinese adults found that the TyG index, along with TyG-BMI, TyG-WHtR, and TyG-WC, was significantly associated with hyperuricemia, with predictive ability stronger in women than in men (23). Among adults with hypertension, the TyG index also demonstrated a linear positive correlation with hyperuricemia, with an odds ratio of 2.39 for hypertensive patients and 2.61 for non-hypertensive participants (24). A cross-sectional study of 42,387 Chinese adults showed that higher TyG levels were associated with an increased risk of hyperuricemia, with risk ratios exceeding those of its two gender components (25). Collectively, these findings suggest that the TyG index can serve as a valuable predictor of hyperuricemia risk across different demographic groups, highlighting the importance of monitoring IR in the prevention and management of hyperuricemia.

Our study identified a non-linear relationship between the TyG index and hyperuricemia in individuals with early-stage CKM syndrome, with a threshold effect observed at a TyG index of 9.50. Interestingly, another study reported a similar non-linear association in a general population, where the inflection point was found at 9.69 (26). These discrepant threshold values can be attributed to multiple factors: Firstly, the metabolic profiles of individuals in early-stage CKM syndrome may differ significantly from those in the general population. Specifically, CKM syndrome is characterized by heightened IR and other metabolic abnormalities that could fundamentally alter the TyG index's predictive capacity for hyperuricemia (27). Moreover, the presence of additional metabolic risk factors in the CKM syndrome population may further exacerbate the impact of the TyG index on hyperuricemia, consequently resulting in a lower threshold compared to the general population. In contrast, Wang et al. explored the nonlinear correlation between the TyG index and hyperuricemia in a hypertensive population using restricted cubic splines. Their results differed markedly, indicating no statistically significant nonlinear relationship between the two (p-nonlinear > 0.05) (24). Collectively, these findings highlight the complexity of interpreting the relationship between the TyG index and hyperuricemia. They underscore the critical importance of considering population-specific characteristics, suggesting that effective risk assessment and management may necessitate tailored approaches across different clinical contexts.

The nonlinear relationship observed between the TyG index and hyperuricemia in early-stage CKM syndrome patients can be attributed to several complex metabolic mechanisms involving threshold-dependent physiological adaptations and compensatory responses. The contrasting associations before and after the inflection point may be explained by the intricate interplay of IR, lipid metabolism, and uric acid regulation through distinct pathophysiological phases.

Prior to the 9.50 threshold, the positive correlation is mediated through multiple interconnected pathways (28–30). Insulin resistance progressively impairs renal uric acid handling by promoting hyperinsulinemia-induced reduction in uric acid clearance through enhanced tubular reabsorption (26, 31). Mechanistically, elevated insulin levels directly stimulate urate transporter 1 (URAT1) expression and suppress ATP-binding cassette subfamily G member 2 (ABCG2) activity, leading to enhanced uric acid reabsorption and reduced secretion in the proximal tubules (32). Furthermore, progressive insulin resistance is associated with accelerated purine synthesis through increased adenosine triphosphate breakdown during elevated triglyceride metabolism, directly contributing to enhanced uric acid production (26). The pro-inflammatory state and oxidative stress associated with increasing insulin resistance create a metabolic environment that promotes uric acid accumulation while simultaneously impairing renal excretory capacity (33, 34).

The critical inflection point at TyG index 9.50 represents a metabolic threshold where compensatory mechanisms begin to predominate over pathological processes. This threshold likely corresponds to a point where the body's adaptive responses to severe insulin resistance reach maximum capacity, triggering protective metabolic adjustments. At this stage, uric acid transitions from its pro-oxidant role to serving as a compensatory antioxidant mechanism against the excessive oxidative stress induced by severe insulin resistance (33). This phenomenon aligns with the concept of metabolic saturation effects, where compensatory mechanisms reach a plateau, thereby attenuating further increases in hyperuricemia risk despite continued insulin resistance progression.

Beyond the 9.50 threshold, several compensatory mechanisms may explain the paradoxical negative association (34–37). Advanced insulin resistance triggers pancreatic β-cell dysfunction, leading to reduced insulin secretion and consequently decreased insulin-mediated uric acid reabsorption (34). Additionally, the glucose-uric acid competitive inhibition mechanism becomes prominent when glucose levels exceed renal threshold, leading to competitive inhibition of uric acid reabsorption and increased uric acid excretion through glucosuria-mediated osmotic diuresis (35). The dual antioxidant-pro-oxidant nature of uric acid suggests that at extreme insulin resistance levels, uric acid may serve a protective role, with homeostatic mechanisms favoring its utilization as an antioxidant buffer against overwhelming oxidative stress (33). However, it is important to acknowledge that the specific mechanisms underlying the paradoxical negative association beyond the 9.50 threshold remain largely hypothetical and require further experimental validation. While the individual components of these proposed mechanisms (β-cell dysfunction, glucose-uric acid competitive inhibition, and uric acid's antioxidant properties) are well-established in the literature, their specific interplay and timing in relation to the observed threshold effect represent a working hypothesis that warrants dedicated mechanistic studies in early-stage CKM syndrome populations.

The identification of this specific threshold value (9.50) in early-stage CKM syndrome populations, compared to the higher threshold (9.69) observed in general populations, suggests that individuals with metabolic dysfunction experience metabolic decompensation at lower insulin resistance levels. This difference may reflect the heightened metabolic vulnerability of CKM syndrome patients, where the presence of additional cardiovascular and renal risk factors creates a lower tolerance threshold for insulin resistance-mediated metabolic disturbances (38, 39). The earlier onset of compensatory mechanisms in this population may represent an adaptive response to prevent further metabolic deterioration, highlighting the clinical significance of this threshold for risk stratification and intervention timing. Future research should focus on mechanistic studies to validate these proposed compensatory pathways and elucidate the precise biological basis for the observed threshold effect, particularly through longitudinal assessments of insulin sensitivity, uric acid metabolism, and renal function in CKM syndrome populations.

We conducted a stratified analysis to examine the differences in the TyG index and hyperuricemia across different subgroups in the early-stage CKM population. Interestingly, the subgroup analysis results based on the forest plot logistic regression showed that the positive correlation between the TyG index and hyperuricemia remained strong, regardless of age, gender, race, smoking, and alcohol consumption. This confirms the reliability and universality of our findings. The consistent association across different eGFR levels (≥60 and <60 ml/min/1.73 m^2^) with nearly identical effect sizes demonstrates the robustness of this relationship independent of baseline kidney function, which is particularly relevant given that renal insufficiency significantly affects uric acid metabolism. Simultaneously, the stratified analysis based on the generalized additive model and smooth curve fitting revealed a U-shaped nonlinear relationship within different subgroups, which further validates the stability of previous research results. Moreover, gender, race, and smoking appeared to influence the association between the TyG index and hyperuricemia in the early-stage CKM population (significant interaction *P* values), while age, drinking status, and eGFR showed no significant interaction effects. The observed interaction effects reflect complex biological mechanisms that warrant detailed mechanistic consideration. Sex-based differences in TyG-hyperuricemia associations are primarily mediated through estrogen's multifaceted metabolic effects. Estrogen enhances uric acid excretion by upregulating organic anion transporters (OAT1, OAT3) expression while downregulating URAT1 activity in renal tubules, promoting renal uric acid clearance (40). Simultaneously, estrogen improves insulin sensitivity through multiple pathways including enhanced GLUT4 translocation, improved mitochondrial biogenesis, and activation of PI3K/Akt signaling cascades (41). Additionally, estrogen's anti-inflammatory properties may attenuate the oxidative stress-mediated link between insulin resistance and hyperuricemia (42). Racial differences likely reflect genetic polymorphisms in key metabolic pathways, including variants in uric acid transporter genes (ABCG2, SLC2A9, SLC22A12) that show significant ethnic distribution differences, with Asian populations showing stronger associations for ABCG2 rs2231142 and different effect sizes for SLC2A9 variants compared to Caucasian populations (43). The smoking-related interactions may result from nicotine's complex effects on insulin sensitivity through increased IRS-1 Ser636 phosphorylation and inflammatory cascades that modify purine metabolism and renal uric acid handling (44).

These mechanistic insights have direct implications for developing individualized prevention strategies in early-stage CKM syndrome management. Sex-specific TyG index thresholds may optimize risk prediction, with potentially higher cut-points for premenopausal women due to estrogen's protective effects on both insulin sensitivity and uric acid excretion (45). Race-specific risk assessment models incorporating both TyG index values and genetic risk profiles could enhance prediction accuracy, particularly beneficial for populations with known genetic predispositions to hyperuricemia or insulin resistance (46). Given the synergistic metabolic effects observed, smoking cessation should be prioritized in CKM syndrome patients with elevated TyG index (47). Furthermore, lifestyle interventions could be tailored based on subgroup characteristics: dietary approaches emphasizing glycemic control may be particularly effective for individuals with genetic predispositions to insulin resistance, while structured exercise programs may show differential effectiveness across sex and ethnic groups (48). These personalized approaches represent a paradigm shift toward precision medicine in early-stage CKM syndrome prevention and management (49).

This study highlights the clinical potential of the TyG index as a simple, cost-effective biomarker for early detection of hyperuricemia in individuals with early-stage CKM syndrome. Unlike traditional markers, the TyG index integrates metabolic and insulin resistance parameters, offering a comprehensive risk assessment tool that could facilitate proactive screening and timely interventions. By identifying a threshold effect, our research provides novel insights that could refine risk stratification strategies, encouraging its inclusion in routine clinical practice and public health guidelines. Clinicians could use the TyG index to guide dietary, lifestyle, or pharmacological interventions targeting metabolic dysfunction and uric acid regulation. Future research should validate these findings in longitudinal studies, investigate underlying mechanisms, and assess the impact of TyG-index-based interventions on long-term outcomes, further solidifying its role in clinical and public health applications.

This study possesses several strengths that enhance its validity and significance. First, the use of a large, nationally representative sample from the NHANES database ensures broad generalizability and robust statistical power. Second, the study focused specifically on individuals with early-stage CKM syndrome, addressing a critical gap in current research by exploring metabolic and uric acid dynamics in this unique population. Third, standardized data collection methods, including biochemical measurements and comprehensive covariate assessments, minimize potential measurement biases and enhance data reliability. Fourth, advanced statistical methods, such as the application of smooth curve fitting and threshold analysis, allowed for the exploration of non-linear relationships and provided nuanced insights into the TyG index's role in predicting hyperuricemia. Finally, rigorous adjustment for a wide range of confounders ensured that the observed associations were as unbiased as possible. Together, these methodological strengths make our findings both credible and impactful, providing a valuable foundation for future research and clinical applications.

This study has several limitations that should be acknowledged. First, due to our inclusion and exclusion criteria, the findings may have limited generalizability. For instance, we excluded individuals under 20 years old, pregnant women, and those with advanced CKM syndrome (stage 4), which means our results cannot be directly applied to these groups. Second, while the NHANES database provides a large, nationally representative sample, the cross-sectional nature of this study restricts our ability to establish causal relationships between the TyG index and hyperuricemia. Third, despite adjusting for multiple confounders, unmeasured or residual confounding cannot be entirely ruled out, as certain factors influencing the relationship, such as genetic predisposition or environmental exposures, may not have been accounted for. Fourth, the study focused primarily on a U.S. population, and while it included diverse ethnic groups, caution is needed when extrapolating the findings to non-U.S. populations or other ethnic groups with distinct metabolic profiles. Finally, we utilized a single measurement of the TyG index and uric acid levels, which may not fully capture their variability over time. Addressing these limitations in future longitudinal and interventional studies could further strengthen the clinical implications of our findings.

## 5 Conclusion

This study is the first to systematically reveal the complex association between the TyG index and hyperuricemia in early-stage CKM syndrome populations. By analyzing 14,716 participants, we confirmed the TyG index as an effective indicator for hyperuricemia risk and uniquely identified its nonlinear relationship's critical inflection point. The results emphasize the significance of TyG index 9.50 as a key threshold for metabolic risk transformation, providing a novel perspective for clinical risk stratification. Future research should explore the physiological mechanisms underlying this nonlinear association, design prospective cohort studies to validate our findings, and develop more precise personalized risk prediction models. Large-scale, cross-ethnic, multi-center studies will help verify our conclusions and provide comprehensive evidence for precision medicine in early-stage CKM syndrome.

## Data Availability

The raw data supporting the conclusions of this article will be made available by the authors, without undue reservation.
