# Supplementary material for: Nonlinear association between triglyceride-glucose index and risk of hyperuricemia in early-stage cardiovascular-kidney-metabolic syndrome: a cross-sectional study of United States population
Source: Front Cardiovasc Med. 2025 Oct 17;12:1553957. doi: 10.3389/fcvm.2025.1553957 (PMC12575291; doi:10.3389/fcvm.2025.1553957)
Supplement: Supplementary file 1 [file Table1.docx]

**Table S1. CKM Syndrome Staging Criteria and Study Population Distribution**

| **CKM Stage** | **Participants (n)** | **Percentage** | **Definition** |
| --- | --- | --- | --- |
| **Stage 0** | 1,384 | 9.4% | No CKM risk factors: Normal BMI (<25 kg/m² or <23 kg/m² if Asian ancestry), normal waist circumference, normoglycemia, normotension, normal lipid profile, and no evidence of CKD or subclinical/clinical CVD |
| **Stage 1** | 3,263 | 22.2% | Excess or dysfunctional adiposity: BMI ≥25 kg/m² (≥23 kg/m² if Asian ancestry) and/or waist circumference ≥88 cm (women)/≥102 cm (men) [≥80 cm (women)/≥90 cm (men) if Asian ancestry], and/or prediabetes, without other metabolic risk factors or CKD |
| **Stage 2** | 9,015 | 61.3% | Metabolic risk factors or CKD: Presence of hypertension, metabolic syndrome, type 2 diabetes, hypertriglyceridemia (≥150 mg/dL), or CKD (eGFR <60 mL/min/1.73 m² or UACR ≥30 mg/g) |
| **Stage 3** | 1,052 | 7.1% | Subclinical CVD: Presence of subclinical atherosclerotic CVD, subclinical heart failure, very high-risk CKD (KDIGO stages G4-G5), or high predicted CVD risk (≥20% 10-year risk) |
| **Total** | **14,714** | **100.0%** | **Early-stage CKM syndrome population** |

**Abbreviations:** BMI, body mass index; CKD, chronic kidney disease; CKM, cardiovascular-kidney-metabolic; CVD, cardiovascular disease; eGFR, estimated glomerular filtration rate; KDIGO, Kidney Disease Improving Global Outcomes; UACR, urine albumin-creatinine ratio.

**Notes:** Study population was limited to early-stage CKM syndrome (stages 0-3). Stage 4 CKM syndrome (clinical CVD or advanced kidney disease) was excluded due to study focus on early disease progression and intervention opportunities. Staging criteria based on AHA 2023 Presidential Advisory classification.
